# High genetic diversity and distinct ancient lineage of Asiatic black bears revealed by non-invasive surveys in the Annapurna Conservation Area, Nepal

**DOI:** 10.1371/journal.pone.0207662

**Published:** 2018-12-05

**Authors:** Rabin Kadariya, Michito Shimozuru, Jesús E. Maldonado, Mohamed Abdallah Mohamed Moustafa, Mariko Sashika, Toshio Tsubota

**Affiliations:** 1 Laboratory of Wildlife Biology and Medicine, Department of Environmental Veterinary Science, Graduate School of Veterinary Medicine, Hokkaido University, Sapporo, Japan; 2 National Trust for Nature Conservation, Khumaltar, Lalitpur, Nepal; 3 Center for Conservation Genomics, Smithsonian Conservation Biology Institute, National Zoological Park, Washington, DC, United States of America; 4 Department of Animal Medicine, Faculty of Veterinary Medicine, South Valley University, Qena, Egypt; National Cheng Kung University, TAIWAN

## Abstract

Asiatic black bears (*Ursus thibetanus*) have a widespread distribution in mountain landscapes, and are considered vulnerable globally, but are low-priority species for conservation in Nepal. Habitat fragmentation, illegal hunting, and human-bear conflict are the major threats to Asiatic black bears across their global range. Having an adequate level of genetic variation in a population helps with adapting to rapidly changing environments, and thus is important for the long-term health of bear populations. Accordingly, we conducted non-invasive surveys of bear populations in the Annapurna Conservation Area (ACA) to elucidate genetic diversity, genetic structure, and the phylogenetic relationship of Asiatic black bears from this region of Nepal to other subspecies. To assess levels of genetic diversity and population genetic structure, we genotyped eight microsatellite loci using 147 samples, identifying 60 individuals in an area of approximately 525 km^2^. We found that the Asiatic black bear population in the ACA has maintained high levels of genetic diversity (H_E_ = 0.76) as compared to other bear populations from range countries. We did not detect a signature of population substructure among sampling localities and this suggests that animals are moving freely across the landscape within the ACA. We also detected a moderate population size that may increase with the availability of suitable habitat in the ACA, so bear-related conflict should be addressed to ensure the long-term viability of this expanding bear populations. Primers specific to bears were designed to amplify a 675 bp fragment of the mitochondrial control region from the collected samples. Three haplotypes were observed from the entire conservation area. The complete mitochondrial genome (16,771 bp), the first obtained from wild populations of the Himalayan black bear (*U*. *t*. *laniger)*, was also sequenced to resolve the phylogenetic relationships of closely related subspecies of Asiatic black bears. The resulting phylogeny indicated that Himalayan black bear populations in Nepal are evolutionary distinct from other known subspecies of Asiatic black bears.

## Introduction

The Asiatic black bear *Ursus thibetanus* has a broad geographic distribution across south and southeast Asia, China, Russia, Korea, and Japan [1]. There are seven recognized subspecies: Japanese black bears (*U*. *t*. *japonicus)* in Japan [2–3], Ussuri black bears (*U*. *t*. *ussuricus)* in far-east Russia, northeast China, and Korea [4–6], Formosan black bears (*U*. *t*. *formosanus*) in Taiwan [7–8], Indochinese/Sichuan black bears (*U*. *t*. *mupinensis*) in southwest China [9], Baluchistan black bears (*U*. *t*. *gedrosianus)* in south Pakistan and Iran, Tibetan black bears (*U*. *t*. *thibetanus)* in the eastern Himalayas and southeast Asia, and Himalayan black bears (*U*. *t*. *laniger*) in the western Himalayas [10–11]. A small population of *U*. *t*. *laniger* is patchily distributed across Pakistan, northwest India, and likely northeast India and Nepal as well [10]. *U*. *t*. *laniger* may overlap with the distribution of *U*. *t*. *thibetanus* across Myanmar and northeast India to possibly Nepal in the Himalayan range. *U*. *t*. *laniger* is distinguished from *U*. *t*. *thibetanus* by its longer and thicker fur with abundant under-wool, and chest marks that are usually smaller and whiter. Recently, evidence for the presence of Himalayan black bears was found using a paw sample collected from a monastery at the Nepal-Tibet border and a fecal sample from a zoo in Pakistan, although the geographic origins of both samples were unknown [11]. Although the range of *U*. *t*. *thibetanus* and *U*. *t*. *laniger* may overlap in Nepal [10], no molecular data from wild populations have been available to date to elucidate the phylogeny of black bears inhabiting the mountainous region of Nepal and throughout their distribution range.

**Fig 1:**
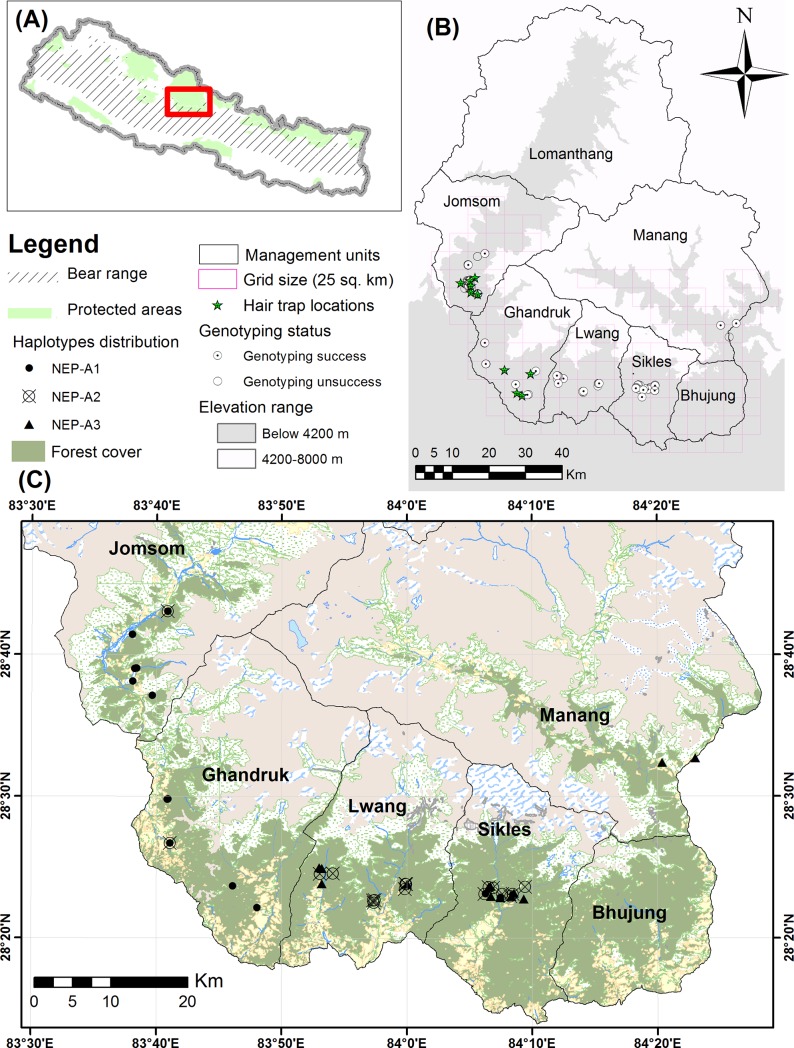
**(A) Distribution range of Himalayan black bears in Nepal (B) Distribution of noninvasive samples with genotyping status and location of hair traps (C) Geographic distribution of three haplotypes in forest habitats of 5 management units.** A total of 147 samples were collected from Annapurna Conservation Area (ACA). The grey color represents maximum elevation range (< 4200 m) of Himalayan black bears except Lomanthang unit. Stars indicate the location of hair traps (n = 11) and white circles with black dots indicate samples (n = 97) which were analyzed for our study. The black circles (n = 24), circles with cross (n = 21) and triangles (n = 15) represent the distribution of control region (CR) haplotypes NEP-A1, NEP-A2 and NEP-A3, respectively. (Data source: Shapefiles and topographical maps, survey department, Government of Nepal, http://nationalgeoportal.gov.np; SRTM DEM, the Earth Explorer, https://earthexplorer.usgs.gov).

In Nepal, black bears are distributed most broadly across mid- to high-elevation mountains (≤ 4200 m), including 13 protected areas (Fig 1A) [12–14]. Recent records showed a range overlap with sloth bears in hilly areas (237 m) in Bardia National Park [13, 15], but no overlap with brown bears (*U*. *arctos pruinosus*) in Nepal [1]. The Asiatic black bear is threatened in much of its habitat by poaching, habitat degradation and fragmentation, and is designated as “vulnerable” in the International Union for Conservation of Nature (IUCN) Red List [1], and as an endangered mammal in Nepal [13]. It is also listed in CITES Appendix I, indicating that this bear species requires comprehensive protection to address the grave conservation threats it faces. Subspecies of Asiatic black bears, including the Himalayan black bears, have not been separately assessed yet in the IUCN Red List. The Asiatic black bear has received little attention in Nepal from the conservation community compared to other charismatic, critically endangered species [13]. Consequently, the status of the black bear population and its genetic characteristics are still poorly understood in Nepal.

Genetic information can be used to better understand the population response of bears to human-induced landscape changes even in the absence of detailed ecological and biological data. Large mammals such as bears often occur at low densities, requiring expansive and continuous landscapes to maintain viable populations [16]. Small, isolated populations are susceptible to extinction through the loss of genetic variation and inbreeding depression [17–18]. Inbreeding depression has been documented in both captive and wild animal populations due to reproduction between genetically related individuals [19–21]. An isolated population may benefit from genetic variants introduced from other populations of the same species [22]. This situation is also applicable for newly recovered bear populations in the mountain landscapes of Nepal [23]. Natural barriers (steep terrain and river) and scattered human settlements have led to habitat fragmentation, which might hinder the movement of bears, impeding gene flow among different populations. The amount of gene flow between bear populations in the forests of the northern high mountains and the adjacent southern middle mountains of Nepal is virtually unknown. Analyzing the genetics of the populations in this region is important for understanding the demographic consequences of habitat isolation and helps to inform the implementation of appropriate conservation action.

Recent records indicate that the wild populations in Nepal are presumably undergoing rapid changes in population size [23–24]. Endangered or vulnerable species not only require a large enough population size, but also sufficient genetic variation for long-term persistence of the species [18, 25]. Considering the conservation and management of endangered species, it is important to evaluate the level of genetic diversity within populations which are supposed to be affected by significant changes in population size. Information on genetic structure and gene flow is crucial for ensuring the sustainability of wild populations. Community-based conservation has been initiated in the Annapurna Conservation Area (ACA) since the late 1980s by Annapurna Conservation Area Project (ACAP) and Conservation Area Management Committees (CAMCs). The conservation awareness, planting of fuelwood species, provision of alternative energy resources, legal prosecution on illegal extraction of forest resources and wildlife poaching, ban on harvesting from standing trees, alternative income generation activities and eco-tourism promotion have been effective in conserving wildlife populations, protecting forest resources, and enhancing livelihoods in local communities [26], but no studies have been conducted on the effect of these conservation measures on the genetic diversity of the local bear population. Non-invasive genetics studies are increasingly being used to monitor the conservation status of Asiatic black bears [27–31], as the direct observation, capture and tagging of bears are difficult and costly in mountain landscapes. This study incorporates both microsatellite markers which are widely used in conservation genetics to quantify genetic diversity, relatedness, population structure and population monitoring, and mitochondrial DNA (mtDNA) sequences which are mainly used to infer phylogenetic relations, evolutionary history and resolving taxonomic issues. This study investigates the present levels of genetic diversity, population structure, and evolutionary relationships of Himalayan black bears in the ACA using both microsatellite markers and mtDNA. We hypothesized that (i) conservation efforts would have a positive effect in the maintenance of genetic diversity of bear populations in the ACA, (ii) the remaining populations of bears inhabiting the northern and southern forests would exhibit genetic substructuring and low levels of geneflow due to the dispersal barriers of high mountains, rivers, habitat fragmentation, and human settlements in the area, and (iii) a distinct lineage of Himalayan black bear (*U*. *t*. *laniger*) inhabits the mountain range in Nepal.

## Materials and methods

### Ethics statement

Hair and fecal samples from bears were collected non-invasively, without capturing or handling any animals. The study permits were granted by the Department of National Parks and Wildlife Conservation (DNPWC), Ministry of Forest and Soil Conservation (MoFSC), Government of Nepal, Kathmandu (Letter No. 490-22/09/2015) and National Trust for Nature Conservation (NTNC)/ACAP, Pokhara, Nepal (Letter No. 546-04/07/2016).

### Study area

This study was carried out in the southern middle and high mountain forests of the ACA (Fig 1B). The ACA, which is bordered in the north by Tibet (China), is a major part of the Chitwan Annapurna landscape, extending from the north to the south in Nepal. It is the first conservation area (7,629 km^2^) established in Nepal, representing 22% of the protected areas in the country. Within the ACA, the forest, which is the preferred habitat of Asiatic black bears, comprise 1,160 km^2^ (15%) from the southern mid-altitude to the north high-altitude mountain region [32]. The forests in the ACA are highly diverse, ranging from subtropical broad-leaved forests to subalpine forests, and mostly consists of *Schima-Castanopsis*, *Pinus roxburghii*, *Quercus* spp., *Acer* spp., *Pinus wallichiana*, *Abies spectabilis*, *Betula utilis*, *Aurndinaria* spp., and *Rhododendron* spp. ACA forests support several endangered species: Himalayan black bears (*U*. *t*. *laniger*), red panda (*Ailurus fulgens*), musk deer (*Moschus chrysogaster*), and clouded leopards (*Neofelis nebulosa*) in the study site and snow leopards (*Panthera unica*), Tibetan brown bears (*U*. *actor pruinosus*), and Himalayan wolves (*Canis lupus chanco*) in the upper Himalayan region. Traditionally, the people of in this region have been highly dependent on natural resources, particularly the mountain forests [33], which have been greatly altered by human migration and tourism activities. The majority of local human inhabitants still rely on the local forest ecosystems for subsistence, for collecting wood, fodder, medicinal plants, and wild foods, for grazing, and as cultural and spiritual sites.

### Sampling

Intensive sampling was conducted in six management units of the ACA during the rainy (July-September) and autumn (October-December) seasons of 2015 and 2016. Four and two of the management units were located in the middle and high mountain regions of the ACA, respectively. We searched for fecal and hair samples within 21 grids (5 × 5 km^2^) over the six units and in the adjoining cropland. We opportunistically collected fecal samples from the agricultural land locating adjacent the bear habitats. The grids were selected based on accessibility, geographic representation, and forest type to better represent the bear population for genetic assessment. We walked along animal trails, villagers’ routes, and mountain ridges to cover the different habitats of bears. If bear signs were observed, the search for fresh feces was intensified as these yield higher-quality DNA [34–35]. The exterior surface of each fresh samples of putative bear feces was rubbed lightly using a cotton swab, which was then stored in a 15-ml vial containing 10 ml of 100% ethanol (Wako, Osaka, Japan) [36–37]. The “freshness” of the fecal sample was estimated based on visual examination and on the external characteristics based on acquired experience of the collector and substantiated by the presence of surroundings bear sign and information from observation of local villagers. Honey-baited hair traps that consisted of a single barbed wire strand with an automated camera (Bushnell corporation) were placed in 11 locations (Fig 1B). A wire, approximately 20 m long, was wrapped around at least 4 trees, 40–50 cm above the ground at each location [29, 38]. Traps were visited every 10 days during a one-month trapping season. We did not detect bear signs at all of the traps, and thus discontinued the placement of hair traps at additional sites. We also collected hair samples, visually identified as bear hairs, from the barbed wire fencing of agricultural land and from the broken branches of trees where bears nested. Hair samples were stored in paper envelopes containing silica desiccant beads. Detailed information about the habitat characteristics, conservation threats and GPS locations (Garmin) were noted for each fecal and hair sample. Disposable latex gloves were replaced after each sample was collected and forceps were immediately rinsed with bleach and 75% ethanol after collecting each hair sample to avoid any possibility of sample cross-contamination.

### Genetic analysis

#### DNA extraction

Genomic DNA was extracted using the Qiagen QIAamp DNA Stool Mini Kit (Qiagen Inc.) for fecal samples and the Wako DNA Extractor FM Kit (Wako, Osaka, Japan) for hair samples according to the manufacturers’ instructions. We included negative controls and processed DNA samples under sterile conditions to avoid cross-contamination.

#### Microsatellite genotyping

For the microsatellite analysis in this study, 27 loci were initially tested using 16 fecal samples to determine genotypes; 11 loci from *U*. *americanus* [39–41], 10 loci from *U*. *arctos* [42], and 6 loci from *U*. *t*. *japonicus* [43]. The primers and combination of these primers for multiplex PCR are listed in S1 Table. Eight loci were finally selected (see result) for further analyses based on the ease of use in multiplex PCR, amplification efficiency, readability and success, having a relatively small (< 0.25) probability of identity (P_ID_), and having a high value (> 0.5) of polymorphic information content (PIC) [29]. We used the *Amelogenin* gene with the primers SE47/SE48 for molecular sexing of individual Asiatic black bears [44–46]. Samples were first multiplexed with the primer sets of three highly variable loci (MU23, G10B and MU50), and then discarded if they did not produce scorable results at any locus. Samples that amplified at all three loci were further amplified using primers for an additional five loci and sex primers. PCR reactions and genotyping were carried out using the conditions described in S1 Text.

#### Genotyping errors

Each sample that was successfully amplified in the first multiplex set of three loci was genotyped two additional times to control for genotyping errors. Poor-quality DNA from fecal samples could lead to the misidentification of individuals and biased estimates [47–48], and so we discarded samples that were not successful in the first round of amplification. If a sample produced a consensus genotype without ambiguous amplifications in both rounds, additional reactions were not carried out. Otherwise, three to four amplifications were conducted for each sample to confirm any allele that was inconsistently scored. A consensus genotype was constructed if at least two replicates were matched in eight loci for each sample; samples missing any locus were excluded from our data set. In some cases, an additional singleplex PCR was performed for the final confirmation of the allele. Maximum likelihood allele dropout (ADO) and false alleles (FA) were calculated from PEDANT version 1.0 with 10,000 search steps for enumerating error [49]. Genotyping errors such as stutter bands, null alleles, and large allele dropouts were verified using MICROCHECKER version 2.2.3 [50].

#### Mitochondrial DNA sequencing

We amplified the left variable region of the mitochondrial control region (CR)/D-loop (approximately 675 bp) of all identified individuals using the bear-specific primer pairs 11H2 and 11L2, and BED 1–2 and BED 3–3 (S3 Table). Amplification conditions and sequencing are described in S1 Text. To resolve the polytomy of phylogenetic relationships of closely-related subspecies of Asiatic black bears based on CR and cytochrome b, whole mitogenome sequencing was performed for two samples that were determined to have different haplotypes for the CR. Bear-specific primers were designed for the amplification of fragments covering the entire mitochondrial genome as in Hirata [51]. In the case of poor PCR performance with these primers, additional primers were also designed to complete the sequencing of each fragment in both directions (S3 Table). Amplification and sequencing were carried out following procedures for CR haplotypes as described in S1 Text.

### Data analysis

#### Genetic diversity and probability of identity

Samples that matched at all eight loci were pooled to create individual genotypes using GIMLET version 1.3.3 [52]. Genetic diversity (mean number of alleles per locus (N_A_), effective no of alleles (N_E_), observed heterozygosity (H_O_), expected heterozygosity (H_E_) and Unbiased expected heterozygosity (u*H*_*E*_*))* and Wright’s inbreeding coefficient (F_IS_) [53] were calculated with GenALEx version 6.5 [54]. The Hardy-Weinberg equilibrium (HWE) of eight microsatellite loci following the exact test and linkage disequilibrium (LD) between all pairs of loci were tested using the web-based program GENEPOP version 4.2 [55]. Bonferroni corrections were applied for multiple comparisons. P_ID_, the probability of identity of siblings (P_ID_Sibs), PIC, and the null allele frequency (Fnull) of each locus, were calculated using CERVUS version 3.0.7 [56]. The GPS coordinates of each genotype and their recaptures were mapped using ArcMap version 10.0 (ESRI, Inc.).

#### Estimation of relatedness and analysis of population genetic structure

Pairwise relatedness values (r) were calculated for all individual bears using the estimator developed by Queller and Goodnight [57] in GenALEx. To determine the patterns of population genetic substructure for the high and middle mountain populations of the ACA, we used a Bayesian clustering analyses in STRUCTURE version 2.3.4 [58]. The same program was also used to detect any genetic structure caused by barriers to bear movement in the conservation area. The range of possible clusters (K) ranged from 1 to 5, and five independent runs were performed with and without prior information of sampling locations. The admixture model with correlated frequencies was run with burn-in periods of 50,000 and 500,000 Markov Chain Monte Carlo (MCMC) iterations. Each individual bear was assigned to a cluster if its membership coefficient (q) was above 0.7, or classified as admixed if q was less than 0.7. To determine the most probable value of K, mean LnProb values as in Pritchard et al. [58] was used as implemented in STRUCTURE HARVESTER [59].

#### Mitochondrial DNA sequencing and phylogenetic inference

Sequences were visually inspected for errors, multiple peaks, and heteroplasmy using FinchTV version 1.4.0 (Geospiza Inc.), and aligned with Clustal W [60] in MEGA 7.0.26 [61]. The exact length of the CR could not be determined due to the presence of a variable number of tandem repeats (VNTRs). The genomic positions of 2 rRNAs, 22 tRNAs, 13 protein-coding genes, and the CR were determined by the whole mitogenome sequence of Asiatic black bears from the Yunnan province of China (GenBank accession no. NC_009971) [62]. All sequences were deposited in the NCBI GenBank database (Accession number MH262297–MH262299 for the CR haplotypes and MH281753 for the complete mitochondrial genome). Haplotype and nucleotide diversity of the CR were analyzed using DnaSP version 6.10.04 [63].

The complete mitogenome and the haplotypes sequences of six subspecies of Asiatic black bears were obtained from the GenBank (S4 Table). The Japanese black bear (Accession no AB863014) [3] was used as an outgroup for the phylogenetic analysis of Asiatic black bears. Insertions and deletions of nucleotides, VNTR sequences of 10-bp units in the CR, and ambiguous sites were excluded from the analyses. Three data sets comprising (1) the left domain (675 bp) of the CR, (2) cytochrome b (1,140 bp), (3) the whole mitogenome except VNTRs of the CR (16,363 bp), and (4) a combination of 12 protein-coding and 2 rRNA genes (12,663 bp) were analyzed. A phylogenetic network tree was constructed using the median joining network [64] in PopART (http://popart.otago.ac.nz) to investigate the possible relationships between CR haplotypes of Asiatic black bears and haplotypes generated from Nepal. Phylogenetic trees were prepared using maximum likelihood (ML) and Bayesian inference (BI) with the programs RAxML version 8.2.10 [65], CIPRES Science Gateway version 3.3, and MrBayes version 3.2.6 [66], respectively. The GTR substitution model (the only model provided in RAxML) was used to infer the maximum likelihood phylogeny using 1,000 bootstrap replicates in RAxML. The best-fit model of nucleotide substitution for BI was selected by jModelTest version 2.1.10 [67], using corrected Akaike information criterion (AICc) values. The Bayesian analysis included two independent runs of four chains for 1,000,000 MCMC generations sampled every 1,000 generations. Nodal support on the BI tree was measured by Bayesian posterior probabilities (BPP). We used Tracer version 1.7 [68] to verify that our effective sample size of the underlying posterior distribution was large enough (> 200) for a meaningful estimation of parameters. Both trees were visualized in FigTree version 1.4.3. Additional genealogical relationships among observed mtDNA haplotypes were inferred using the neighbor-joining method [69] in the program MEGA, with the genetic distance between haplotypes calculated using Kimura’s two parametric method [70]. No gamma correction was made for the difference in substitution rates of different pairs of nucleotides, as the observed sequence divergence was too low to have a significant effect on estimates [71]. A maximum parsimony analysis was also conducted in MEGA for comparison.

## Results

### Non-invasive sampling and genotyping of microsatellite loci

A total of 126 fecal and 21 hair samples were collected in five management units in the ACA from elevations ranging from 1,725 to 3,580 m. Of 126 fecal samples, 44 and 82 were collected during the rainy and autumn seasons, respectively. Twenty-four samples that did not produce usable DNA extracts, and 26 additional samples that amplified inconsistently for several loci or did not yield complete genotypes even after the third round of PCR were removed from further analyses (Table 1). Amplification was unsuccessful at two loci (MSUT5 and MSUT6) for 16 fecal samples while following the original annealing temperature with slight adjustment to the protocol. Four loci (G1A, MU51, MU09, and MU59) amplified for some samples only whereas the remaining 21 loci were successfully amplified for all samples. All 25 loci were polymorphic, ranging from 2 (G10X) to 7 (MU50) alleles each. Three loci (G10P, G1A and G10B) deviated from HWE, but the differences were not statistically significant after Bonferroni corrections. Two loci (MSUT4 and G10X) had higher P_ID_ (> 0.250) and lower PIC (< 0.5) values than the other loci. Following amplification, we selected 8 of the 19 polymorphic loci with high amplification success, also taking into account their ease of use in multiplex PCR and readability of their allelic peaks (S2 Table). We then successfully genotyped these eight microsatellites using at least two PCR experiments from 73% (n = 92) of the fecal samples and 24% (n = 5) of the hair samples. The low rate of genotyping success from hair samples was possibly due to the poor quality of DNA extracts from samples that were old. We had an overall genotyping success of 66% (n = 97) for both hair and fecal samples (Table 1) with 0–4% allelic dropout errors, with negligible presence of false alleles. The Micro-checker program did not detect any errors due to stutter bands, large allele dropouts or deficits of heterozygotes. The proportion of successfully amplified fecal samples collected in autumn (84%) was noticeably higher than that of samples collected during the rainy season (52%).

**Table 1:** Details of non-invasive sample size, genotyping success and the number of individuals located in forest vs. farmland in each management unit.

Management Unit	Total sample^†^	Genotype status					Individual location	
		DNA unsuccess	Ambiguous	Success (no)	Success (%)	Unique	Forest	Farmland
Jomsom	56(41)	18(8)	16(15)	22(18)	39(44)	10(10)*	1	9
Ghandruk	22(17)	5(1)	5(5)	12(11)	55(65)	10(9)	7	3
Lwang	29 (29)	1(1)	1(1)	27(27)	93 (93)	20(20)	19	1
Sikles	35(34)		2(1)	33(33)	94(97)	18(18)	18	1^#^
Manang	5 (5)		2(2)	3(3)	60(60)	2(2)		2
**Total**	**147(126)**	**24(10)**	**26(24)**	**97(92)**	**66(73)**	**60(59)**	**45**	**16**

^**†**^Sixty one samples (16 hairs and 45 feces) were collected from the agricultural land.

*One identical genotype represented by both feces and hair sample.

#One individual found both in farmland and forest.

Numbers in parentheses were represented by feces only.

### Genetic variability and individual distribution

The number of alleles (N_A_) for the eight microsatellite loci ranged from 5 to 10, and averaged 7.6, which was higher than the effective numbers of alleles (4.4). MU23 and MU50 were highly polymorphic with 10 alleles whereas G10B and UamB5 were the least polymorphic with 5 alleles (Table 2). In the Jomsom unit, an individual with seven recaptures had a base-pair difference mutation in MU23 (S5 Table). We could not confirm whether this was due to a historical mutation in the population or a mutation specific to this individual. Combined across all loci, the average values of observed (H_O_) and expected (H_E_) heterozygosity were 0.798 and 0.760, respectively. No significant deviation from HWE (P > 0.306) or LD was detected between pairs of the eight microsatellite loci (P > 0.07). Three pairs had slight deviations that were not statistically significant after Bonferroni corrections (P > 0.02). The cumulative P_ID_ and P_ID_Sibs were 3.17 × 10^−09^ and 5.5 × 10^−04^, respectively, which indicated that the power of the markers selected was more than sufficient to discriminate among individuals. Similarly, PIC averaged 0.727, and ranged from 0.617 to 0.816. The value of F_IS_ was -0.041, indicating no signs of inbreeding among populations. The negative value of F_IS_ also indicates an excess of heterozygous individuals in the region, a finding supported by the lower proportion (< 0.1) of null alleles.

**Table 2:** Genetic variability parameters for the 8 microsatellite loci used to evaluate the population of Annapurna Conservation Area, Nepal (N = 60 individuals).

Locus	Multiplex	N_A_	N_E_	H_O_	H_E_	uH_E_	PIC	P_ID_	P_ID_Sibs	F_IS_	P	Fnull	ADO
MU23	MP1	10	5.00	0.833	0.800	0.807	0.772	0.069	0.367	-0.033	0.759	-0.02	0.0
G10B	MP1	5	3.59	0.650	0.721	0.727	0.684	0.115	0.418	0.107	0.112	0.06	0.0
MU50	MP1	10	5.23	0.883	0.809	0.816	0.784	0.062	0.361	-0.084	0.941	-0.05	0.6
G10C	MP2	9	6.11	0.917	0.836	0.843	0.816	0.047	0.344	-0.088	0.986	-0.04	0.0
MU05	MP2	8	4.31	0.917	0.768	0.775	0.736	0.086	0.387	-0.185	1.000	-0.10	0.0
MU61	MP2	8	5.17	0.800	0.807	0.813	0.780	0.064	0.363	0.017	0.353	0.01	3.8
UamB5	MP3	5	2.95	0.717	0.662	0.667	0.617	0.160	0.459	-0.075	0.785	-0.04	0.0
UamD2	MP3	6	3.13	0.667	0.680	0.686	0.627	0.155	0.449	0.029	0.306	0.01	1.6
Mean/Cum		7.63	4.44	0.798	0.760	0.767	0.727	3.17X10^-9^	5.5X10^-4^	-0.041			
SE		0.73	0.40	0.038	0.023	0.023							

N_A_, observed no of allele; N_E_, effective no of alleles; H_O_, observed heterozygosity; H_E_, expected heterozygosity; uH_E_, unbiased expected heterozygosity; PIC, polymorphic information content; P_ID_, probability of identity (locus); P_ID_Sibs, probability of siblings identity (locus); F_IS_, Wright's inbreeding coefficient; P, P values for exact tests of Hardy-Weinberg equilibrium (level of significance, α = 0.05); Fnull, predicted frequency of null alleles; ADO, allele drop out (%); MP, multiplex primer; SE, standard error.

From the 97 samples that were successfully genotyped, we identified 60 individuals, of which 20 were sampled multiple times (S5 Table). Two individuals were detected up to seven times, while 40 individuals (67%) were recorded only once during the sampling period. Sex determination identified 32 (54%) males and 28 (46%) females. Most of the bears (n = 48) were recorded in three management units in the southern forest during multiple surveys. Similarly, ten and two individuals were recorded in two high mountain management units located in Mustang and Manang districts, respectively. In total, 60 individuals were found in approximately 525 km^2^ (11.5 individuals/100 km^2^) of the ACA that included both forests and surrounding farmlands.

### Genetic relatedness and population structure

**Fig 2:**
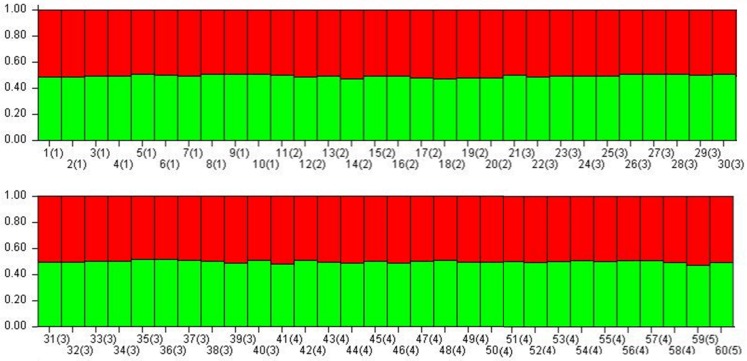
Population structure of Himalayan black bears from ACA, Nepal without prior sampling information. Vertical bar represented individual bears and color represented membership coefficient (q) of each individual which was less than 0.7 when K = 2; (1) = Jomsom, (2) = Ghandruk, (3) = Lwang, (4) = Sikes, (5) = Manang management units, and no 1–60 represents the individual identified from microsatellite analysis; Similar patterns were observed with prior information of location model.

The average pairwise relatedness value for 1,770 pairs among 60 individuals was less than 0.05 (r = -0.017, SE = 0.005), which suggests that individuals were highly unrelated to one another. Models with and without LOCPRIOR showed that no individual was assigned with high posterior probability (q ≥ 0.7) to any of the two clusters at K = 2 (Fig 2). Individuals from the ACA never formed a separate cluster even with increasing values of K. Results from STRUCTURE and the log probability of all individuals revealed that the value of K is most likely 1 (S1 Fig). High variance between iterations and large standard deviations were observed with other estimated values of K (S6 Table). These results indicated that black bears from the ACA effectively constituted a single randomly-mating population without subdivision and that all individuals were highly admixed.

### Sequence characteristics, haplotypes in the mitochondrial DNA control region, and geographic distribution of haplotypes

For all 60 individuals, the left domain of the CR (675 bp) was successfully sequenced. We sequenced three haplotypes that were distinguished by two transition substitutions of adenine (A) and guanine (G) and a repeated-number variation at a T-repeat site (Table 3). Haplotypes NEP-A1 (n = 24) and NEP-A2 (n = 21) were widely distributed in the study area from the northwest to south while NEP-A3 (n = 15) was detected in the northeast to the south of the ACA. All three haplotypes were found in the middle of the southern area of the ACA (Fig 1C). The overall haplotype diversity (H) and nucleotide diversity (π) from the CR were 0.381 (± 0.057) and 0.113% (± 0.017%), respectively, which were calculated by excluding the T-repeat motif. Haplotype and nucleotide diversity are relatively low (H < 0.5 and π < 0.1%) considering only the population of the ACA. This study produced first mitogenome (16,771 bp) sequenced from natural populations of *U*. *t*. *laniger* using hair and fecal samples, although a mitogenome of *U*. *t*. *laniger* was previously sequenced from an old skin found in a monastery near the Nepal-Tibet border [11]. VNTRs located in the CR were too long to be sequenced directly to represent the actual length of the mitogenome. The K2P distances among Asiatic black bears calculated using MEGA ranged from 0.8 to 5.6% for cytochrome b (average 2.5%), 1.6 to 7.4% for the CR (average 3.4%), 0.8 to 3.8% (average 1.9%) for the complete data set except VNTRs of the CR, and 0.7 to 3.7% for the 12 protein-coding and 2 rRNA genes (average 1.9%). Compared to sequences from Nepal, sequences from Japanese black bears showed higher divergence, while sequences from black bears on the main Asian continent showed less divergence with the exception of those from *U*. *t*. *mupinensis* (S7 Table).

**Table 3:** Variable positions and observed frequencies of the left domain of the CR for the 3 haplotypes Himalayan black bears from ACA in Nepal.

Haplotypes	Used length	Position no^#^			Population			Individuals	
		105	166	558	NW	S	NE	No	Percentage
NEP–A1	675	T	G	A	9	15	0	24	40
NEP–A2	674	–	.	.	1	20	0	21	35
NEP–A3	674	–	A	G	0	13	2	15	25

NW, northwest (Jomsom); S, south (Ghandruk, Lwang, and Sikles); NE, northeast (Manang).

^#^The positions were numbered for a haplotype NEP-A1 from the 5’ end of sequence. Dot indicate identity with the nucleotides of NEP-A1. Dash indicates variation in the number of Ts at a T-repeat site (98–105 for NEP-A1).

### Phylogenetic relationships

**Fig 3:**
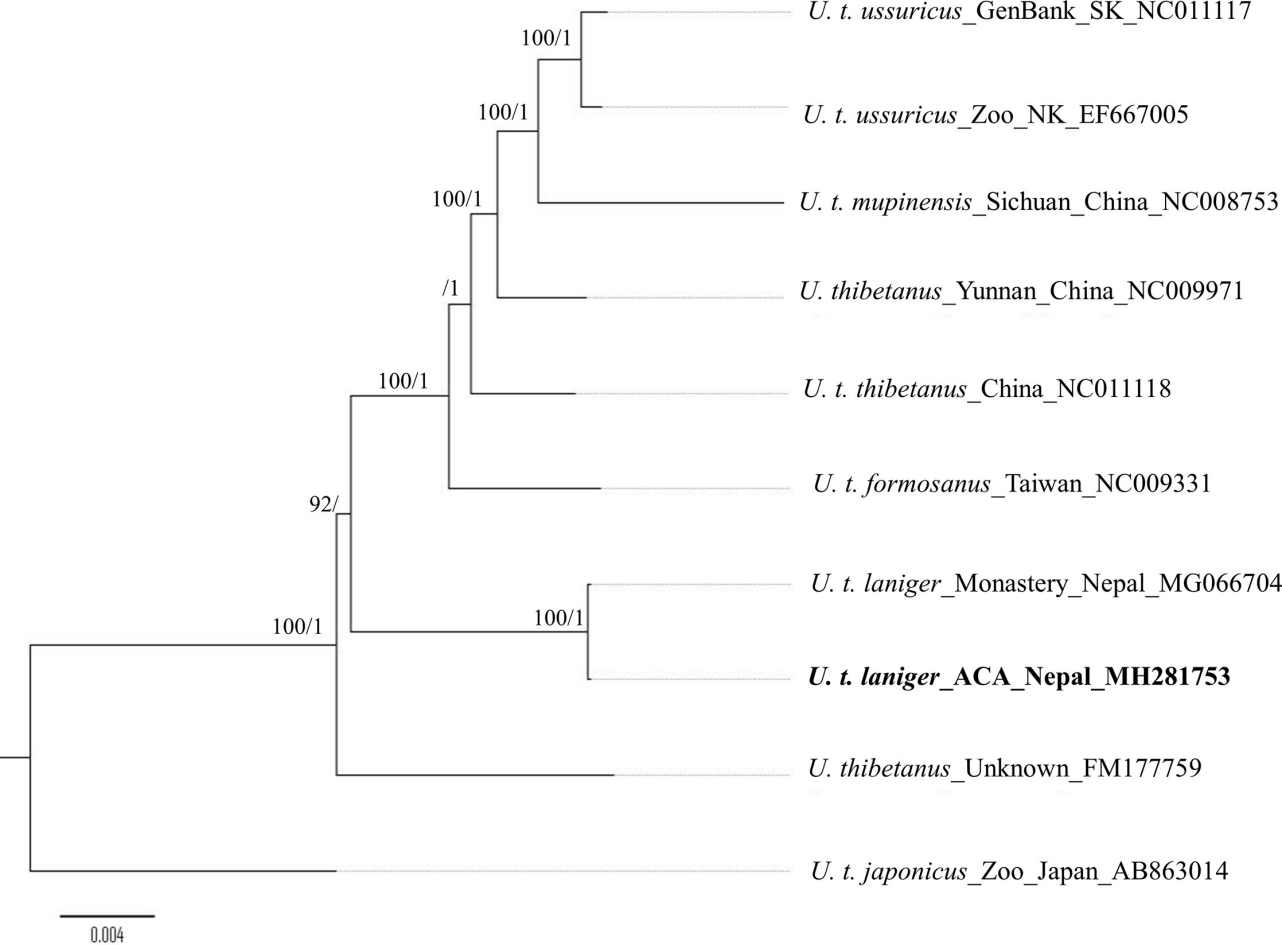
Phylogenetic relationships among Asiatic black bears using mitogenomes excluding the VNTR’s of the CR (16,363 bp). The number at the nodes of the branches indicate bootstrap supporting values in percentage (BP) and Bayesian posterior probabilities (BPP) based on maximum likelihood and Bayesian analyses, respectively. Identical tree topology was also obtained from 12 mitochondrial protein-coding genes and two rRNA genes (12,663 bp). The mtDNA sequence of *U*. *t*. *japonicus* (AB863014) was used as an outgroup. Only bootstrap values over 75% and BPP over 0.95 are shown. Sequences are identified by the subspecies name, origin and country name, followed by the GenBank accession number (NK, North Korea; SK, South Korea). The position of mitogenome haplotypes from *U*. *t*. *laniger* generated in this study is shown in bold face.

**Fig 4:**
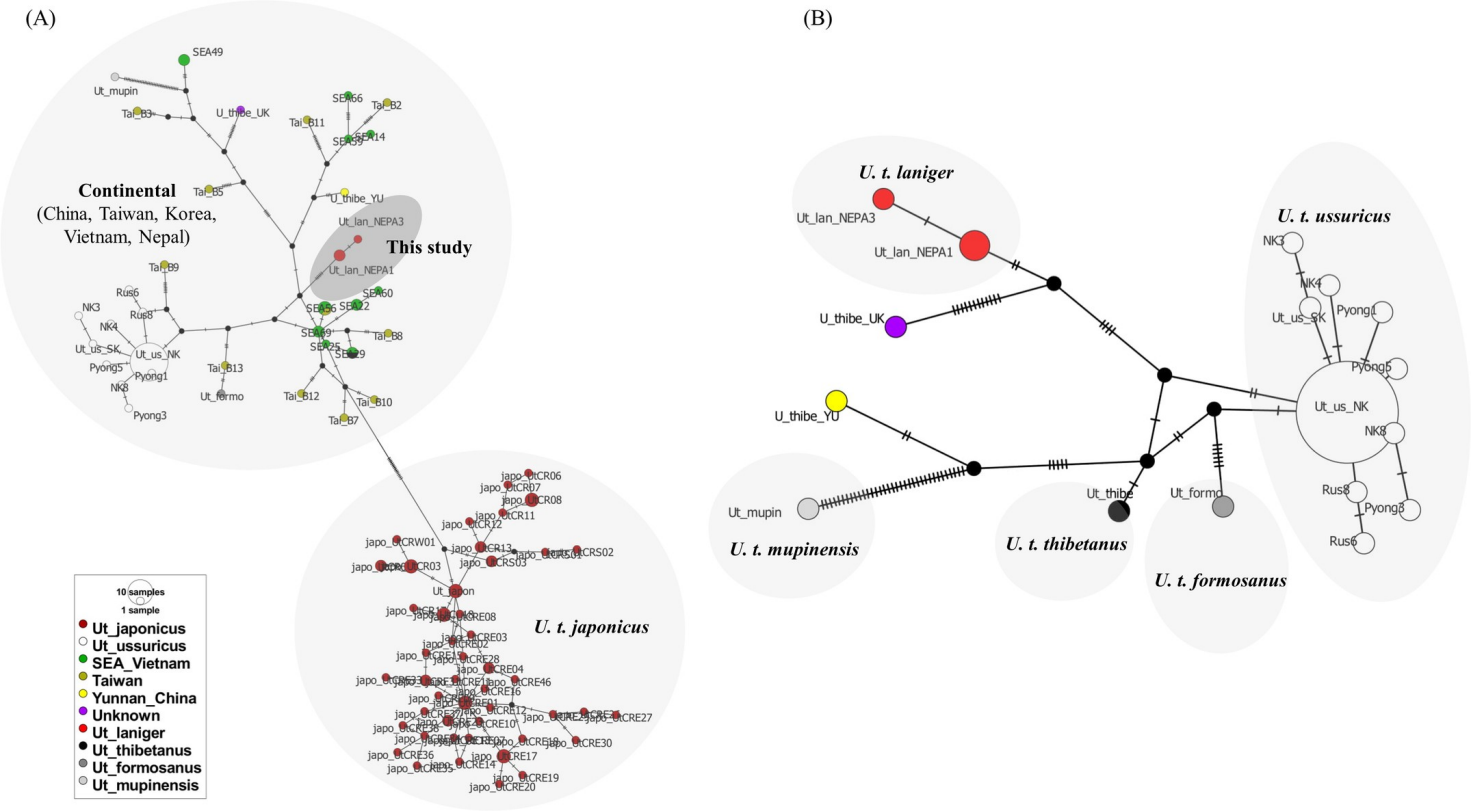
Median joining network based on CR haplotypes by using POPART. A total of 523 positions were included in the final analysis after complete deletion. Network tree was generated using all published haplotypes for 6 subspecies of Asiatic black bears, unknown haplotypes from southeast Asia (Vietnam), Taiwan, Yunnan province of China and unknown origin; (A) for 130 haplotypes of Asiatic black bears and (B) based on continental subspecies of 40 haplotypes after excluding the haplotypes from Japan, Taiwan zoo and Vietnam’s rescue center. The detail information of each haplotypes is presented in S4 Table.

The GTR+G model was selected as the best model using the AICc approach in jModelTest 2 for cytochrome b, genes in the complete dataset and protein-coding genes, while the HKY+I model was selected for the CR. Maximum likelihood and Bayesian phylogenetic analyses revealed that *U*. *t*. *laniger* from the ACA of Nepal formed a monophyletic clade with *U*. *t*. *thibetanus* and with respect to other reported Asiatic black bear subspecies. A similar tree topology was obtained using mitogenomes (excluding the VNTRs of the CR) and the dataset of 12 protein-coding and 2 rRNA genes (Fig 3). Identical well-resolved and strongly-supported tree topologies (BP 92–100% and BPP 1) consistent with previous studies were obtained in both maximum likelihood and Bayesian analyses using mitogenomes of the Asiatic black bear subspecies [3, 11]. However, tree topologies obtained using the cytochrome b gene and the left domain of the CR did not resolve the phylogeny of *U*. *thibetanus* subspecies (S2 Fig), likely because of homoplasy caused by the high substitution rate in this region, and the lack of phylogenetically informative sites [3]. Therefore, we used a median joining network, which has been demonstrated to better visualize intraspecific sequence variation in the CR. In the network, the haplotypes from the ACA of Nepal were grouped separately from *U*. *t*. *thibetanus* and other subspecies (Fig 4). Although unknown source of Asiatic black bear mitogenome (FM177759) contributed to minor differences in phylogenetic resolution, overall, haplotypes of *U*. *t*. *laniger* formed a clade basal to all other Asiatic black bears subspecies on the Asian continent in analyses using different methods and sequence sets. Neighbor-joining and maximum parsimony analyses of cytochrome b sequences produced similar topologies to those generated with mitogenomes. However, the basal placement of haplotypes of *U*. *t*. *laniger* had weak bootstrap support (S3 Fig), while the topology based on the CR had weak or no bootstrap support.

## Discussion

### Genetic diversity and population structure of Himalayan black bears in Nepal

This study revealed that there is no any evidence to suggest that the genetic diversity and geneflow of Himalayan black bears in Annapurna region of Nepal are inadequate, and we did not detect any evidence of inbreeding in the population. We found no significant differences between observed and expected heterozygosity, low levels of relatedness among bear individuals, and a lack of population substructure. The average expected heterozygosity was similar to that found in Indian (0.72 [31]) and Russian (0.65–0.76 [28, 72]) bear populations, and higher than the heterozygosity found in Japanese (0.66 [27]; 0.68 [73]; 0.53–0.70 [74]) and North Korean (0.67) populations [72]. No other studies are available for comparisons among broad geographic regions in Nepal. The ACA is located in the central part of a continuous mountain landscape [75] and the genetic diversity in the ACA could represent the high levels of connectivity at a wide scale among black bear populations in Nepal. The estimated genetic diversity of black bears in the ACA is similar to those of populations of North American bears in continuous habits, such as the population of brown bear (*U*. *arctos*) in Kluane [76]. As these North American populations are regarded as stable [77], the genetic diversity of black bears in the ACA may be sufficient to maintain a stable population.

No evidence of population substructure was found among the ACA populations, indicating a high degree of admixture throughout the conservation area. Our results are similar to those reported for the population in Dachigam National Park in northwest India [31]. The results may also be due to a large proportion of the genomes of bears in the ACA being admixed ancestrally. Our study found substantial evidence for gene flow between the high mountain and the southern middle mountain forest regions, suggesting that bears in this conservation area are panmictic, freely moving long distances between regions and breeding effectively in both areas. Male-biased dispersal and female-biased philopatry are known in most mammalian species [78]. As reported in the American black bear [79] and Himalayan black bear [31], male black bears have a larger home range and disperse further than females. The lack of genetic substructure within our dataset indicates that bears have no effective barriers to movement within the ACA, although we expected signatures of at least two subpopulations between the northwest and southern region of the ACA, due to the potential migration barriers of rivers, high elevation mountains, and human settlements in this area. Our results showed that either these landscape features have not restricted the movement of bears between the northwest and southern regions or, alternatively, it could be that there has been a more recent restriction of movement between these regions but not enough time has elapsed to leave a genetic signature of population structure. This also provides further support for population connectivity within the neighboring protected areas via contiguous forest and highlights their importance in the overall management of the species. It is highly recommended that further genetic studies in other protected areas be carried out using the molecular methods developed here to evaluate admixing ancestry and population substructuring over a broader geographic range. Additional factors such as forest cover, food availability, human impact, road networks, and other human infrastructure can be assessed in relation to the movement of bears [80].

Globally, habitat fragmentation and degradation are major causes of population decline and species extinction [81], which also affect genetic variation and population viability. A fragmented landscape means that individuals have to expend more effort to move through their habitat, which potentially decreases overall movement and genetic exchange [80]. During the last few decades, middle mountain forests have been well managed in Nepal, resulting in increased forest cover due mainly to the community forestry program (e.g. plantation, control on grazing and on illegal extraction of forest resources, regulations for forest product collection) throughout the regions [75, 82] and the community-based conservation program in the ACA [26]. The improved forest conditions include increased habitat connectivity, which may have played a crucial role in the maintenance of genetic diversity in bear populations here, by facilitating the free movement of the Himalayan black bears. However, the construction of roads and hydropower facilities, increasing human-bear conflict, and retaliatory killing of bears are emerging threats for bear populations in the mountainous habitats of Nepal.

Available data indicate that there are approximately 500 black bear individuals in Nepal [13]. From this study, a minimum of 60 individuals is estimated to inhabit a single conservation area based on non-invasively collected samples. The estimated minimum density of black bears in the ACA (11.5 bears/100 km^2^) was less than previous estimates from the national parks of the western Himalayas (17 bears/100 km^2^) and Thailand (8–29 bears/100 km^2^) [83–84], which could not be extrapolated to estimate the bear population outside protected areas. Bear densities from studies in Russia and Japan were estimated to be 8–11 bears/100 km^2^ and 8–9 bears/100 km^2^, respectively [85]. Our analysis showed that 25% (n = 16) of the individual bears sampled were recorded from agricultural land, demonstrating the potential for human-bear conflict in the rainy season. Proper management to reduce crop losses to bears will help secure the future survival of bear populations and maintain their genetic diversity in the long-term.

Sampling in this study was mainly opportunistic and geographically biased, and individual bears were not found repeatedly due to the large geographic distances. Although collecting fecal samples from agricultural land and the nearby bamboo forest during the rainy season was relatively manageable, it was difficult to obtain high-quality DNA due to the high levels of moisture in the samples [86]. We recommend that future fecal sampling be conducted during the dry season [37]. In addition, all individuals, which may include cubs, were included in our calculations. In future studies, a standardized capture-recapture approach may help to improve the dataset and estimate a more precise population size. Future systematic surveys conducted throughout the potential distribution range of black bears will likely reveal that the total population is much higher in ACA. In general, our results support the ACA as being a highly suitable habitat for bears, and also support the potential for the bear population to increase in the future.

### Phylogenetic relationships of Asiatic black bears

This is the first study using genetic and geographic data from the mitogenome sequences from six subspecies and two Asiatic black bears of unknown origin. The only subspecies that was not represented in our phylogeny was *U*. *t*. *gedrosinus*. In previous studies, the lack of phylogenetic resolution for Asiatic black bears was primarily due to the low level of variation in the much shorter sequences of cytochrome b or the CR. Previous studies also placed the Japanese subspecies (*U*. *t*. *japonicus*) as the basal group to all other *U*. *thibetanus* subspecie*s* [3, 87]. In our phylogenetic analysis, the individual FM177759 [88] branched off more basally than the clade of *U*. *t laniger*. However, the geographical origin of this individual is unknown. Based on the largest mtDNA dataset available from Asiatic black bears, our mitogenome-based phylogeny provides strong evidence that *U*. *t*. *laniger* is the most basal of all black bear subspecies on the Asian continent. The Japanese black bear was estimated to have diverged from continental black bears approximately 1.48 Mya [3] and the ancestor of *U*. *t*. *laniger* diverged from other lineages of *U*. *thibetanus* at 475 Ka BP [11]. Individuals of the northeast Asian *U*. *t*. *ussuricus* also clustered into a single clade [6, 72].

Our mitogenome analysis strongly supports previous finding of a monophyletic lineage of *U*. *t*. *laniger* in the ACA that is considered ancient with respect to all other mainland Asiatic black bears subspecies [3, 11] and does not support the results of Choi et al. [6] that places *U*. *t*. *mupinensis* as basal to all other *U*. *thibetanus* in their Clade A. Although the sequences used in Choi et al. [6] were from the same mitogenome regions used in our analysis, the *U*. *t*. *mupinensis* in our phylogenetic tree was placed monophyletic to the other subspecies of *U*. *thibetanus* but the *U*. *t*. *laniger* sequences were the basal group. Our phylogenetic analyses inferred from mitogenomes of two unknown and six known subspecies suggesting that whole mitogenomes (without the VNTRs of the CR) were more informative and provide a high resolution for Asiatic black bear phylogeny. The CR and cytochrome b genes are not sufficiently informative to resolve phylogenetic relationships in Asiatic black bears, particularly among such closely related subspecies. Although the left domain of CR is considered the most appropriate region to use in constructing phylogenetic trees [5], we did not obtain sufficient resolution in our analysis. Additional caution is needed when analyzing data from samples of unknown origin as interbreeding between *U*. *t*. *japnonicus* and *U*. *t*. *ussuricus* from China [87], and *U*. *thibetanus* and *U*. *malayanus* in northern Cambodia [89] have been reported. This may affect the interpretation of results from phylogenetic analyses of samples from these regions. Samples of southeast Asiatic black bears from Vietnam and Taiwan were taken from a rescue center and a zoo, respectively. Phylogenetic analyses that included these samples produced complex topologies, suggesting a complicated structuring of populations in these regions [72].

Our phylogenetic analyses demonstrated that lineages of the Himalayan black bears in Nepal are ancient and genetically distinct from lineages of other subspecies of Asiatic black bears from the main continent (China, Taiwan and southeast Asia). Our results corroborate the findings of previous studies of *U*. *t*. *laniger* from the Himalayan region based on the specimen from the Mustang district monastery [11]. Mitogenomes from the samples NEP-A1 and NEP-A2 were similar and closely related to mitogenome haplotype derived from the sample collected at the monastery, which is located far away from the black bears’ natural habitat and outside the known distribution of Asiatic black bears. The T-repeat region and VNTRs of the CR were excluded from the original sequence (MG066704) reported in GenBank, so we were unable to compare the T-repeat region of the haplotypes of NEP-A1 and NEP-A2 with monastery sample. The bear skin at the monastery could have originated from the southern part of the district, which is highly populated by Himalayan black bears based on our microsatellite analysis. These three novel haplotypes came from samples collected from the wild mountain habitats where black bears were never recorded as being reintroduced. Individuals of *U*. *t*. *laniger* from India and Pakistan also formed a well-supported clade with other continental bear subspecies using the amplicon sequence of cytochrome b [11].

Based on our results, we propose that *U*. *t*. *laniger* is likely distributed throughout the western Himalayan mountain range from Nepal to Pakistan, including the northwest India. Further sampling along the eastern Himalayas in Nepal, India, Bhutan, and Myanmar may help to more clearly define the distribution range of *U*. *t*. *laniger* and *U*. *t*. *thibetanus* in the east. We found no evidence of a habitat overlap between the Tibetan brown bear (*U*. *a*. *pruinosus*), the sloth bear (*U*. *ursinus*), and *U*. *t*. *laniger* in ACA. The brown bear has been sporadically recorded in the northern part of the ACA and sloth bears inhabit southern Chitwan National Park of the Chitwan-Annapurna landscape [13].

## Conclusions

Our intraspecific phylogeny demonstrates that the wild population of Himalayan black bears (*U*. *t*. *laniger*) from Nepal is genetically distinct and belongs to an ancient lineage of continental Asiatic black bears. We were also able to count the minimum number of bears in the ACA population from non-invasive samples. The genetic analyses revealed that no management interventions are currently needed to maintain genetic diversity in the population, but human-bear conflict has to be addressed for long-term survival of the bears. Our study indicates that community-based conservation in the ACA may have contributed to the viability of the bear population here, with low levels of population substructuring. Still, our results were based on samples collected within a single conservation area. Additional non-invasive studies on evolutionary lineage, population status, genetic diversity, and genetic structure should be conducted over the entire mountain landscape of Nepal with sufficient sample replicates to represent this widespread species. This is the first genetic assessment of Asiatic black bears in Nepal and the information herein should be incorporated in Nepal’s national assessment, and when revising the IUCN status and conservation action plan for Asiatic black bears.

## Supporting information

S1 Text Genetic analyses of nuclear and mitochondrial DNA. (DOCX)

S1 Table Microsatellite loci and primers tested for genotyping fecal and hair samples collected non-invasively for the Himalayan black bears in Annapurna Conservation Area, Nepal. (DOCX)

S2 Table Characteristics of microsatellite markers tested in the present study. Analysis were carried out based on 16 fecal samples which later identified 8 unique genotypes.
(DOCX)

S3 Table Primers designed for mitochondrial genome and control region (CR) sequencing. (DOCX)

S4 Table Overview of the sequence/haplotypes used for the phylogenetic analysis. (XLSX)

S5 Table Database of 60 Himalayan black bears for 8 microsatellites in 5 management units of Annapurna Conservation Area, Nepal. (XLSX)

S6 Table Summary and raw STRUCTURE HARVESTER output for K = 1 to K = 5 local population of ACA. Each K was performed 5 iterations and a mean value was estimated. The lowest value of Ln likelihood and the low variance of Ln likelihood suggest that the true value of K = 1, implying that Himalayan back bears of ACA are one breeding population and that there is gene flow between northern high mountains and southern mid hills.
(XLSX)

S7 Table Evolutionary distances among *U*. *thibetanus* for control region (CR), cytochrome b and mitogenome of mtDNA sequences. (XLSX)

S1 Fig Structure results of 60 individuals from 5 different management units of ACA. The mean of estimated Ln probability of data is higher when population sub cluster K = 1. Y axis values are fixed from -1600 to -1670 for clear presentation of graph.
(DOCX)

S2 Fig Phylogenetic relationship of Asiatic black bears based on the nucleotide sequence of CR (675 bp). The numbers at the nodes of the branches indicate bootstrap supporting values in percentage (BP) and Bayesian posterior probabilities (BPP) based on maximum likelihood and Bayesian analyses, respectively. The mtDNA sequence of *U*. *t*. *japonicus* (AB863014) was used as an outgroup. Only bootstrap values over 50% and BPP over 0.5 are shown. Sequences are identified by the subspecies name, origin and country name, followed by the GenBank accession number (NK, North Korea; SK, South Korea). The position of mitogenome haplotypes from *U*. *t*. *laniger* generated in this study is shown in bold face.
(DOCX)

S3 Fig Phylogenetic tree based on the complete cytochrome b gene (1,140 bp). The numbers denoted at the node are the bootstrap values based on Neighbor joining/Maximum parsimony methods, respectively. Only the values greater than 50% are shown. Sequences are identified by the subspecies name, origin and country name, followed by the GenBank accession number (NK, North Korea; SK, South Korea). The mitogenome generated in this study is marked in bold face.
(DOCX)
