# Supplementary material for: High genetic diversity and distinct ancient lineage of Asiatic black bears revealed by non-invasive surveys in the Annapurna Conservation Area, Nepal
Source: PLoS One. 2018 Dec 5;13(12):e0207662. doi: 10.1371/journal.pone.0207662 (PMC6281213; doi:10.1371/journal.pone.0207662)
Supplement: S1 Text — (DOCX) [file pone.0207662.s001.docx]

**S1 Text. Genetic analyses of nuclear and mitochondrial DNA.**

**Microsatellite genotyping**

Using TaKaRa Multiplex PCR Assay Kit (TaKaRa Bio Inc., Shiga, Japan), PCR reactions were carried out in 3 multiplex reactions, each in a final volume of 15 μl, containing 7.5 μl of 2x multiplex PCR buffer (Mg^2+^, dNTP plus), 75 nl of multiplex PCR enzyme mix, 0.50 μl of primer mix of each 7.5 μM, 1 μl of DNA extract and remaining RNase-free water. Amplification was performed in Applied Biosystems Veriti Thermal Cycler (Applied Biosystems, USA) with an initial denaturation at 94 ºC for 1 minute, followed by 40 cycles of 94 ºC for 30 seconds, 50-55 ºC (S2 Table) for 1 minute, 72 ºC for 1 minute, and a final extension at 72 ºC for 10 minutes. PCR products were visualized on ABI Prism 310 Genetic Analyzer using the GeneScan software. Following PCR, products were diluted 1:50-1:80, and 1 μl was mixed with an internal lane size standard according to the manufacturer’s instructions (GeneScan-500 LIZ Size Standard, Applied Biosystems, USA). Microsatellite allele sizes were estimated by comparison to size standard using GeneMapper version 4.1 (Applied Biosystems, USA).

**MtDNA analysis**

The PCR amplification was carried out in 25 µl reaction volume which contained 2.5 µl of 10X *Ex Taq* Buffer, 2 µl of dNTP Mixture, 125 nl of *Ex Taq* polymerase (TaKaRa Bio Inc., Shiga, Japan), 0.5 µl each of 10 µM forward (11H2) and reverse (11L2) primers, 1 µl of DNA template and remaining PCR-grade water. After denaturation at 94 ºC for 1 minute, 35 cycles were performed as 94 ºC for 30 seconds, 55 ºC for 30 seconds, 72 ºC for 90 seconds, and a final extension at 72 ºC for 10 minutes. Five microliter of PCR aliquots were run on a 1.5% agarose gel and visualized by ethidium bromide staining under ultraviolet illumination to confirm the amplification of targeted region by primers. Nested PCR was conducted by using internal primer pair (BED 1-2 & BED 3-3) following the same procedures only for those fragments which were not amplified by the first primer pair. PCR products were purified using FastGene Gel/PCR Extraction Kit (NIPPON Genetics Co. Ltd), measured DNA concentration using a spectrometer (NanoDrop 2000; Thermo scientific) and sequenced in both directions using same amplify primers with an ABI 3730 XL DNA Analyzer following the manufacture’s protocol. Amplification and sequencing were repeated in case of ambiguous results.
