# Supplementary material for: High genetic diversity and distinct ancient lineage of Asiatic black bears revealed by non-invasive surveys in the Annapurna Conservation Area, Nepal
Source: PLoS One. 2018 Dec 5;13(12):e0207662. doi: 10.1371/journal.pone.0207662 (PMC6281213; doi:10.1371/journal.pone.0207662)
Supplement: S1 Table — (DOCX) [file pone.0207662.s002.docx]

**S1 Table. Microsatellite loci and primers tested for genotyping fecal and hair samples collected noninvasively for the Himalayan black bears in Annapurna Conservation Area, Nepal.**

| **Locus** | **Multiplex** | **Labelling dye** |  | **Primer sequence (5'-3')**  **Forward (F)/Reverse(R)** | **Ta** | **Repeat motif** | **References** |
| --- | --- | --- | --- | --- | --- | --- | --- |
| G1A | MP2 | FAM | F: | GACCCTGCATACTCTCCTCTGATG | 55 | (GT)19.5 | Paetkau et al. 1995 |
|  |  |  | R: | GCACTGTCCTTGCGTAGAAGTGAC |  |  |  |
| G10B | MP1 | VIC | F: | GCCTTTTAATGTTCTGTTGAATTTG | 55 | (GT)21 | Paetkau et al. 1995 |
|  |  |  | R: | GACAAATCACAGAAACCTCCATCC |  |  |  |
| G10C | MP3 | NED | F: | AAAGCAGAAGGCCTTGATTTCCTG | 55 | (GT)21.5 | Paetkau et al. 1995 |
|  |  |  | R: | GGGGACATAAACACCGAGACAGC |  |  |  |
| G1D | MP5 | VIC | F: | GATCTGTGGGTTTATAGGTTACA | 55 | (GT)17.5 | Paetkau et al. 1995 |
|  |  |  | R: | CTACTCTTCCTACTCTTTAAGAG |  |  |  |
| G10J | MP8 | PET | F: | GATCAGATATTTTCAGCTTT | 55 | (GT)19 | Paetkau et al. 1998 |
|  |  |  | R: | AACCCCTCACACTCCACTTC |  |  |  |
| G10L | MP7 | NED | F: | GTACTGATTTAATTCACATTTCCC | 55 | (GT)34 | Paetkau et al. 1995 |
|  |  |  | R: | GAAGATACAGAAACCTACCCATGC |  |  |  |
| G10M† | MP4 | FAM | F: | TTCCCCTCATCGTAGGTTGTA | 55 | (GT)21 | Paetkau et al. 1995 |
|  |  |  | R: | TTTCCAAATAATTTAAATGCATCC |  |  |  |
| G10P | MP3 | VIC | F: | AGGAGGAAGAAAGATGGAAAAC | 55 | (GT)21 | Paetkau et al. 1995 |
|  |  |  | R: | TCATGTGGGGAAATACTCTGAA |  |  |  |
| G10X | MP3 | FAM* | F: | CCACCTTCTTCCAATTCTC | 55 | (GT)20.5 | Paetkau et al. 1998 |
|  |  |  | R: | TCAGTTATCTGTGAAATCAAAA |  |  |  |
| MU05 | MP2 | VIC* | F: | GTGATTTTTCTTGTAGCCTAGG | 55 | (TG)17 | Taberlet et al. 1997 |
|  |  |  | R: | GAAACTTGTTATGGGAACCA |  |  |  |
| MU09 | MP4 | VIC* | F: | TTGAAGTTCAGGGTAAATGC | 55 | (TG)19 | Taberlet et al. 1997 |
|  |  |  | R: | ATATAGCAGCATATTTTTGGCT |  |  |  |
| MU10 | MP5 | NED | F: | TTCAGATTTCATCAGTTTGAC | 55 | (TG)23 | Taberlet et al. 1997 |
|  |  |  | R: | CAGCATAGTTACACAAATCTCC |  |  |  |
| MU23 | MP1 | NED | F: | GCCTGTGTGCTATTTTATCC | 55 | (TG)19 | Taberlet et al. 1997 |
|  |  |  | R: | AATGGGTTTCTTGTTTAATTAC |  |  |  |
| MU26 | MP7 | VIC | F: | GCCTCAAATGACAAGATTTC | 55 | (TG)19 | Taberlet et al. 1997 |
|  |  |  | R: | TCAATTAAAATAGGAAGCAGC |  |  |  |
| MU50 | MP1 | FAM | F: | TCTCTGTCATTTCCCCATC | 55 | (TG)24 | Taberlet et al. 1997 |
|  |  |  | R: | AAAGGCAATGCAGATATTGT |  |  |  |
| MU51 | MP2 | NED | F: | GCCAGAATCCTAAGAGACCT | 55 | (CA)19 | Taberlet et al. 1997 |
|  |  |  | R: | AAGAGAAGGGACAGGAGGTA |  |  |  |
| MU59 | MP4 | NED | F: | GCTCCTTTGGGACATTGTAA | 55 | (TG)22 | Taberlet et al. 1997 |
|  |  |  | R: | GACTGTCACCAGCAGGAG |  |  |  |
| MU61 | MP5 | FAM | F: | ACCCAGAGAAGTCCGATTAC | 55 | (CA)19 | Taberlet et al. 1997 |
|  |  |  | R: | CTGCTACCTTTCATCAGCAT |  |  |  |
| MU64 | MP8 | NED | F: | ACTCAACACAACCATTAAATCA | 55 | (GT)18 | Taberlet et al. 1997 |
|  |  |  | R: | AGGACCCAAATGACACTACA |  |  |  |
| UamB5 | MP6 | NED | F: | CCGGTGGATCTATCTCAGAGT | 55 | CATC | Meredith et al. 2009 |
|  |  |  | R: | GGGATCTTGTCTATCCTGCTC |  |  |  |
| UamD2 | MP6 | VIC | F: | ACACCTGTCTTCCCTTCCTAAC | 55 | TAGA | Meredith et al. 2009 |
|  |  |  | R: | TTCCATCTGAGAGGCTGAAC |  |  |  |
| MSUT2 | MP9N | PET | F: | AGTGAATCCTAAACAGGTTA | 55 | (AC)20 | Kitahara et al. 2000 |
|  |  |  | R: | TAATATGAATATGGTGTGCT |  |  |  |
| MSUT4 | MP10 | VIC | F: | GTGTCCAACTGTAGATGA | 50 | (TG)14 | Kitahara et al. 2000 |
|  |  |  | R: | TGAGTAATATTCTTTTCTCT |  |  |  |
| MSUT5 | MP9 | VIC | F: | GGGACTGAGCCTCTCATC | 50/55 | (TC)15 | Kitahara et al. 2000 |
|  |  |  | R: | TCCAATATTTTGTCTGAGTG |  |  |  |
| MSUT6 | MP9 | FAM | F: | CATATGGTGACTAAGATAAC | 50/55 | (TG)20 | Kitahara et al. 2000 |
|  |  |  | R: | AAGAGATGATTTCTGTCTC |  |  |  |
| MSUT7 | MP10 | NED | F: | TGGAAAATATTCTCATTC | 50 | (CA)3(CA)3(CA)13 | Kitahara et al. 2000 |
|  |  |  | R: | TTGTAGGTTACTGGTTAC |  |  |  |
| MSUT8 | MP9N | FAM | F: | GATCCTGGGACTTCTCAG | 55 | (TC)3 (TC)5GT)11 | Kitahara et al. 2000 |
|  |  |  | R: | TCCAGAGAAAGAGGACTG |  |  |  |
| Amelogenin | MP6 | PET | F: | CAGCCAAACCTCCCTCTGC | 55 |  | Yamamoto et al. 2002 |
|  |  |  | R: | CCCGCTTGGTCTTGTCTGTTGC |  |  |  |

Ta, annealing temperature (^º^C)

^†^Primer sequence was modified based on GenBank #U22089.

^*^Reverse primers were labelled with a fluorescence dye.
