# Supplementary material for: High genetic diversity and distinct ancient lineage of Asiatic black bears revealed by non-invasive surveys in the Annapurna Conservation Area, Nepal
Source: PLoS One. 2018 Dec 5;13(12):e0207662. doi: 10.1371/journal.pone.0207662 (PMC6281213; doi:10.1371/journal.pone.0207662)
Supplement: S2 Table — Analysis were carried out based on 16 fecal samples which later identified 8 unique genotypes. (DOCX) [file pone.0207662.s003.docx]

**S2 Table. Characteristics of microsatellite markers tested in the present study.** Analysis were carried out based on 16 fecal samples which later identified 8 unique genotypes.

| **Multiplex**  **set** | **Locus** | **Labelling dye** | **Ta** | **Allele size** | **N_A_** | **Amplification efficiency*** | **Amplification readability*** | **Amplification success (%)** | **H_O_** | **H_E_** | **PIC** | **P_ID_** | **P_ID_Sibs** | **P** |
| --- | --- | --- | --- | --- | --- | --- | --- | --- | --- | --- | --- | --- | --- | --- |
| MP1 | **MU50** | FAM | 55 | 212-224 | 7 | good | good | 100% | 0.875 | 0.817 | 0.735 | 0.086 | 0.389 | 0.867 |
|  | **G10B** | VIC | 55 | 154-166 | 5 | good | good | 100% | 0.500 | 0.800 | 0.711 | 0.102 | 0.400 | 0.037 |
|  | **MU23** | NED | 55 | 110-124 | 6 | good | good | 100% | 0.875 | 0.858 | 0.776 | 0.067 | 0.364 | 0.424 |
| MP2 | G1A | FAM | 55 | 192-196 | 3 | Good | good | 44% | 0.000 | 0.714 | 0.555 | 0.211 | 0.490 | 0.031 |
|  | **MU05** | VIC | 55 | 136-146 | 6 | good | good | 100% | 0.875 | 0.817 | 0.730 | 0.090 | 0.390 | 0.842 |
|  | MU51 | NED | 55 | 108-122 | 4 | fair | good | 63% | 0.625 | 0.617 | 0.510 | 0.246 | 0.523 | 0.610 |
| MP3 | G10X | FAM | 55 | 186-188 | 2 | fair | good | 100% | 0.375 | 0.325 | 0.258 | 0.530 | 0.730 | 1.000 |
|  | G10P | VIC | 55 | 152-160 | 4 | good | good | 100% | 0.250 | 0.758 | 0.658 | 0.136 | 0.429 | 0.003 |
|  | **G10C** | NED | 55 | 102-118 | 6 | good | good | 100% | 1.000 | 0.850 | 0.766 | 0.072 | 0.370 | 1.000 |
| MP4 | G10M | FAM | 55 | 204-210 | 4 | good | good | 100% | 0.625 | 0.592 | 0.510 | 0.243 | 0.533 | 0.778 |
|  | MU09 | VIC | 55 | 130-148 | 5 | good | good | 81% | 1.000 | 0.817 | 0.727 | 0.094 | 0.391 | 1.000 |
|  | MU59 | NED | 55 | 106-130 | 6 | fair | good | 88% | 1.000 | 0.842 | 0.759 | 0.075 | 0.374 | 1.000 |
| MP5 | **MU61** | FAM | 55 | 201-219 | 6 | good | good | 100% | 0.875 | 0.850 | 0.766 | 0.072 | 0.370 | 0.742 |
|  | G1D | VIC | 55 | 180-186 | 3 | good | good | 100% | 0.500 | 0.633 | 0.511 | 0.248 | 0.515 | 0.270 |
|  | MU10 | NED | 55 | 122-128 | 4 | good | fair | 100% | 0.500 | 0.725 | 0.618 | 0.164 | 0.451 | 0.097 |
| MP6 | **UamD2** | VIC | 55 | 206-226 | 5 | good | good | 100% | 0.750 | 0.792 | 0.701 | 0.108 | 0.406 | 0.456 |
|  | **UamB5** | NED | 55 | 144-160 | 4 | good | good | 100% | 1.000 | 0.692 | 0.592 | 0.180 | 0.471 | 1.000 |
| MP7 | MU26 | VIC | 55 | 190-210 | 4 | good | good | 100% | 0.750 | 0.700 | 0.605 | 0.169 | 0.464 | 0.796 |
|  | G10L | NED | 55 | 124-142 | 6 | good | good | 100% | 0.625 | 0.775 | 0.691 | 0.110 | 0.414 | 0.181 |
| MP8 | MU64 | FAM | 55 | 191-203 | 5 | good | good | 100% | 0.750 | 0.700 | 0.595 | 0.180 | 0.467 | 0.731 |
|  | G10J | VIC | 55 | 98-112 | 6 | good | good | 100% | 0.625 | 0.733 | 0.654 | 0.131 | 0.439 | 0.350 |
| MP9N | MSUT8 | FAM | 55 | 114-124 | 4 | good | good | 100% | 1.000 | 0.758 | 0.658 | 0.136 | 0.429 | 1.000 |
|  | MSUT2 | PET | 55 | 77-99 | 6 | good | good | 100% | 0.750 | 0.833 | 0.748 | 0.081 | 0.380 | 0.131 |
| MP10N | MSUT7 | NED | 50 | 118-138 | 4 | good | good | 100% | 0.625 | 0.692 | 0.582 | 0.191 | 0.473 | 0.488 |
|  | MSUT4 | VIC | 50 | 92-102 | 5 | good | good | 100% | 0.500 | 0.533 | 0.474 | 0.276 | 0.569 | 0.608 |
| Mean/combined | |  |  |  | 4.8 |  |  |  |  | 0.729 | 0.636 | 3.53x10^-22^ | 1.45x10^-09^ |  |

Ta, annealing temperature ^º^C; N_A_, number of alleles; H_O_, observed heterozygosity; H_E_, expected heterozygosity; PIC, polymorphic information content; P_ID_, probability of identity; P_ID_Sibs, probability of identity of siblings; P, P values for exact test of Hardy-Weinberg equilibrium (level of significance, α = 0.05).

^*^Amplification efficiency and readability were estimated following Uno et al. 2012.

The highlighted loci were selected for amplification of all samples.
