# Supplementary material for: High genetic diversity and distinct ancient lineage of Asiatic black bears revealed by non-invasive surveys in the Annapurna Conservation Area, Nepal
Source: PLoS One. 2018 Dec 5;13(12):e0207662. doi: 10.1371/journal.pone.0207662 (PMC6281213; doi:10.1371/journal.pone.0207662)
Supplement: S3 Table — (DOCX) [file pone.0207662.s004.docx]

**S3 Table. Primers designed for mitochondrial genome and control region (CR) sequencing.**

| mtDNA fragment | Prime name^a^ |  | Primer sequence (5'-3')  Forward (F)/Reverse(R) | Position^b^ | References |
| --- | --- | --- | --- | --- | --- |
| Amplifying primers | | | | | |
| 1 | mtDNA_1H | F: | CAAATGGGACATCTCGATGGACTA | 16754 | [90] |
|  | mtDNA_1L | R: | CAGCTATCACCAGGCTCGTTAG | 2347 | [90] |
| 2 | mtDNA_2AH_2 | F: | GAGAAAGTACCGCAAGGGAAC | 2105 | This study |
|  | mtDNA_2BL_2 | R: | CACCCTAACAAAGCCCTGTC | 3582 | This study |
| 3 | mtDNA_3AH | F: | AGCAATCCAGGTCGGTTTCTATC | 3416 | [51] |
|  | mtDNA_3BL | R: | GTGAGCGATTGAAGAGTATGCTAG | 5373 | [51] |
| 4 | mtDNA_4H_2 | F: | AAGTCACACAAGGCGTTCCT | 5165 | This study |
|  | mtDNA_4L_2 | R: | GACAGTCCACCCAGTTCCTG | 6626 | This study |
| 5 | mtDNA_5H_2 | F: | GCTCTCAGCCTTTTGATTCG | 6336 | This study |
|  | mtDNA_5L_3 | R: | TTGATTCGTGCCGAACTAGG | 6348 | This study |
| 6 | mtDNA_6AH_2 | F: | TTCCGACTATCCAGATGCCTAC | 7562 | This study |
|  | mtDNA_6BL | R: | TAGGCCTGAATGAGGGCTAC | 9479 | [51] |
| 7 | mtDNA_7H_2 | F: | TTGGCTCACTTTCTACCTCAAG | 9217 | This study |
|  | mtDNA_7BL | R: | GCTGATAGGGAGTCGGTAAAG | 11271 | [51] |
| 8 | mtDNA_8H_2 | F: | CTGCGAAGCAGCACTAGGAC | 10997 | This study |
|  | mtDNA_8L_2 | R: | TGCTGACTGCGAAAGCATAG | 12801 | This study |
| 9 | mtDNA_9H_2 | F: | AGCATCAAGCCATCCTTCAC | 12356 | This study |
|  | mtDNA_9L_2 | R: | TGGCGGTCTCGATAATTAGG | 13857 | This study |
| 10_1 | mtDNA_10H_2 | F: | AAGATTATTGCCTTCTCCACCTC | 13557 | This study |
|  | mtDNA_10L_2 | R: | TGCTGGGAGATCAATGAGTG | 15133 | This study |
| 10_2 | mtDNA_10BH_2 | F: | AAAACCCCACAAAACTCATCAC | 14931 | This study |
|  | mtDNA_10CL_2 | R: | TGCTTCTTCCTTGAGTCTTGG | 16287 | This study |
| 11 | mtDNA_11H_2^c^ | F: | ATGAATCGGAGGACAACCAG | 16072 | This study |
|  | mtDNA_11L_2^c^ | R: | AGCTACATAAACGCGGTTGG | 902 | This study |
| CR | mtDNA_BED1-2^c^ | F: | TACCCTCCCCAAGACTCAAG | 16271 | This study |
| CR | mtDNA_BED3-3^c^ | R: | GGAAAAATCAATAGGAGGGAGAC | 823 | This study |
| Sequencing primers | | | | | |
| 1 | mtDNA_1_seq1 | R: | GATTAGCAAGGGGTGGTGAG | 1573 | This study |
| 3 | mtDNA_3_seq1 | F: | CCGTAGCCCAGACAATTTCA | 4054 | This study |
| 6 | mtDNA_6_seq1 | F: | AATTGCACTCCCATCGCTAC | 8151 | This study |
| 7 | mtDNA_7_seq1 | F: | CCTGAACCCGCTAGAAGTACC | 9893 | This study |
| 8 | mtDNA_8_seq1 | R: | TTGATGGTAGGAGGGAGTGC | 12204 | This study |
| 10 | mtDNA_10_seq1 | F: | CGTCTCCAACCAGAAAGGAC | 14387 | This study |

^a^The letter codes H and L refer to the heavy and light strands of the mitochondrial DNA respectively, and the name was given following the primer name of Hirata [51].

^b^ Position corresponds to nucleotide numbers of Asiatic black bears (NC009971) from Yunnan province of China [62].

^c^These region represented the amplification of CR haplotypes.

Amplifying and sequencing primers were used for direct sequencing of the purified PCR product.

**References**

**[51]** Hirata D, Mano T, Abramov AV, Baryshnikov GF, Kosintsev PA, Vorobiev AA, et al. Molecular phylogeography of the brown bear (*Ursus arctos*) in Northeastern Asia based on analyses of complete mitochondrial DNA sequences. [Mol Biol Evol.](https://www.ncbi.nlm.nih.gov/pubmed/?term=Molecular+Phylogeography+of+the+Brown+Bear+(Ursus+arctos)+in+Northeastern+Asia+Based+on+Analyses+of+Complete+Mitochondrial+DNA+Sequences) 2013;30(7):1644-52. doi: 10.1093/molbev/mst077.

**[62]** Yu L, Li YW, Ryder OA, Zhang YP. Analysis of complete mitochondrial genome sequences increases phylogenetic resolution of bears (Ursidae), a mammalian family that experienced rapid speciation. BMC Evol Biol. 2007;7:198. doi: [10.1186/1471-2148-7-198](https://doi.org/10.1186/1471-2148-7-198)

**[90]** Delisle I, Strobeck C. Conserved primers for rapid sequencing of the complete mitochondrial genome from carnivores, applied to three species of bears. Molecular Biology and Evolution. 2002;19(3):357–361. doi: [10.1093/oxfordjournals.molbev.a004090](https://doi.org/10.1093/oxfordjournals.molbev.a004090)
