# Supplementary material for: High genetic diversity and distinct ancient lineage of Asiatic black bears revealed by non-invasive surveys in the Annapurna Conservation Area, Nepal
Source: PLoS One. 2018 Dec 5;13(12):e0207662. doi: 10.1371/journal.pone.0207662 (PMC6281213; doi:10.1371/journal.pone.0207662)
Supplement: S1 Fig — The mean of estimated Ln probability of data is higher when population sub cluster K = 1. Y axis values are fixed from -1600 to -1670 for clear presentation of graph. (DOCX) [file pone.0207662.s009.docx]

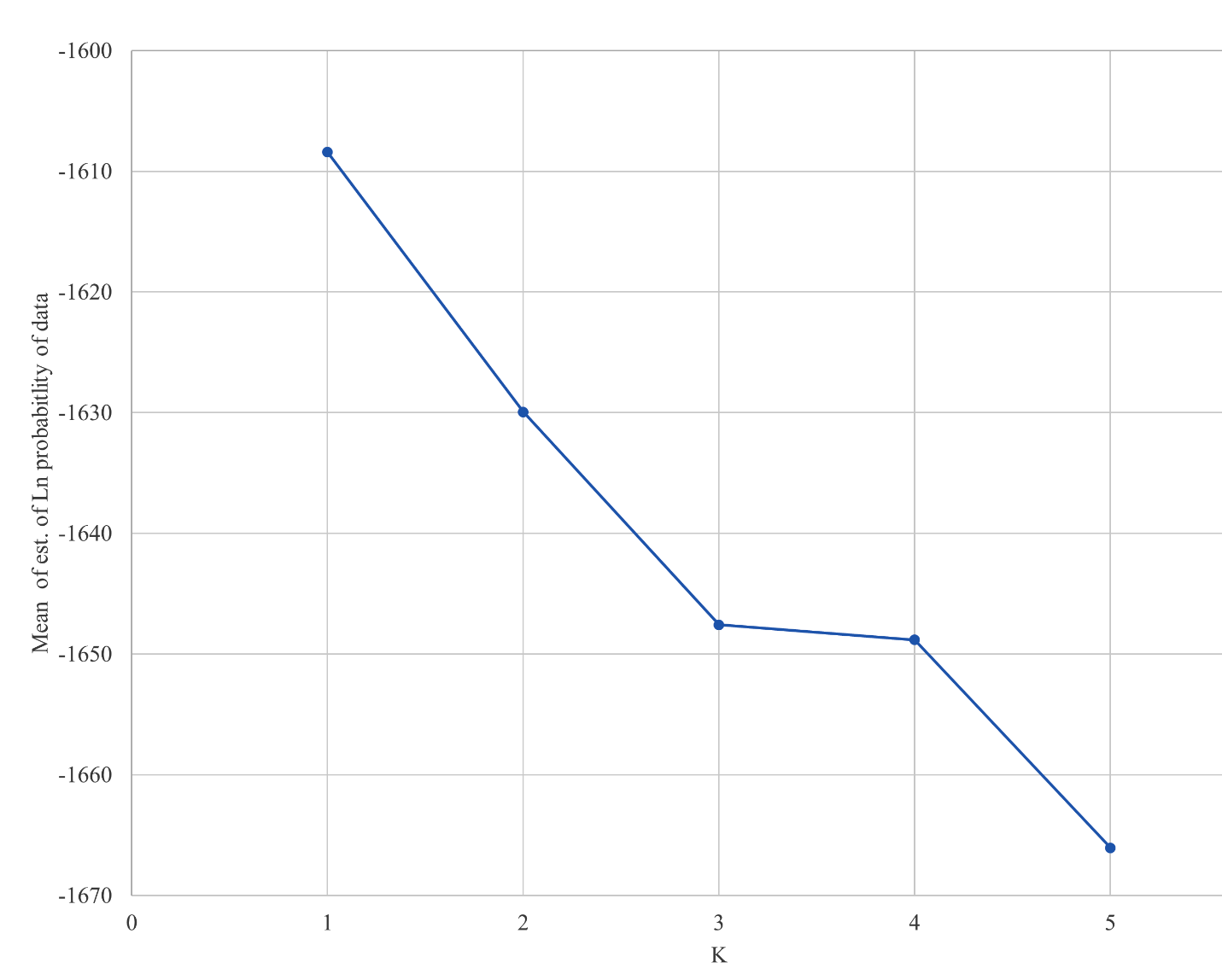


**S1 Fig. Structure results of 60 individuals from 5 different management units of ACA.** The mean of estimated Ln probability of data is higher when population sub cluster K = 1. Y axis values are fixed from -1600 to -1670 for clear presentation of graph.
