# Supplementary material for: High genetic diversity and distinct ancient lineage of Asiatic black bears revealed by non-invasive surveys in the Annapurna Conservation Area, Nepal
Source: PLoS One. 2018 Dec 5;13(12):e0207662. doi: 10.1371/journal.pone.0207662 (PMC6281213; doi:10.1371/journal.pone.0207662)
Supplement: S2 Fig — The numbers at the nodes of the branches indicate bootstrap supporting values in percentage (BP) and Bayesian posterior probabilities (BPP) based on maximum likelihood and Bayesian analyses, respectively. The mtDNA sequence of U. t. japonicus (AB863014) was used as an outgroup. Only bootstrap values over 50% and BPP over 0.5 are shown. Sequences are identified by the subspecies name, origin and country name, followed by the GenBank accession number (NK, North Korea; SK, South Korea). The position of mitogenome haplotypes from U. t. laniger generated in this study is shown in bold face. (DOCX) [file pone.0207662.s010.docx]

**
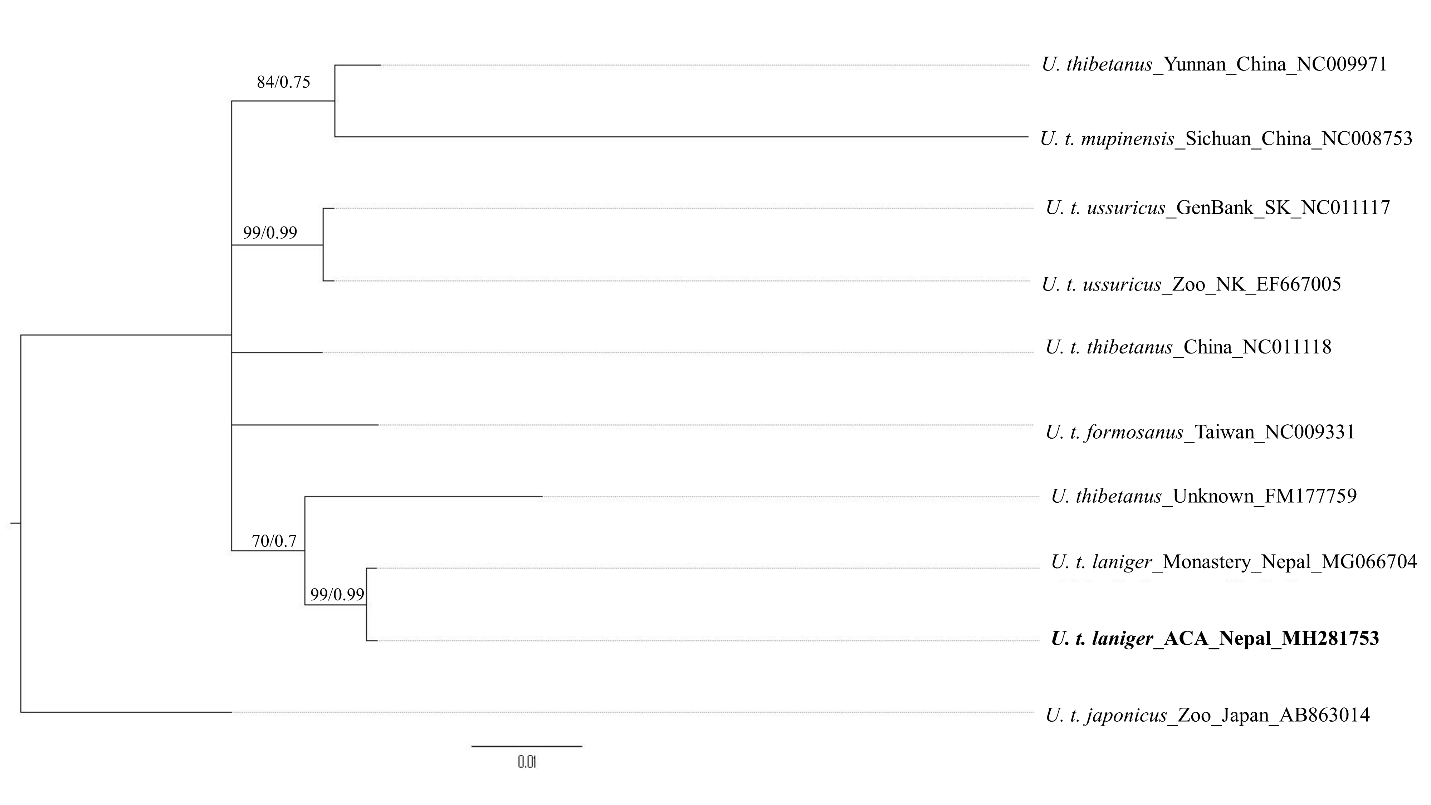
**

**S2 Fig. Phylogenetic relationship of Asiatic black bears based on the nucleotide sequence of CR (675 bp).** The numbers at the nodes of the branches indicate bootstrap supporting values in percentage (BP) and Bayesian posterior probabilities (BPP) based on maximum likelihood and Bayesian analyses, respectively. The mtDNA sequence of *U. t. japonicus (*AB863014*)* was used as an outgroup. Only bootstrap values over 50% and BPP over 0.5 are shown. Sequences are identified by the subspecies name, origin and country name, followed by the GenBank accession number (NK, North Korea; SK, South Korea). The position of mitogenome haplotypes from *U. t. laniger* generated in this study is shown in bold face.
