# Supplementary material for: High genetic diversity and distinct ancient lineage of Asiatic black bears revealed by non-invasive surveys in the Annapurna Conservation Area, Nepal
Source: PLoS One. 2018 Dec 5;13(12):e0207662. doi: 10.1371/journal.pone.0207662 (PMC6281213; doi:10.1371/journal.pone.0207662)
Supplement: S3 Fig — The numbers denoted at the node are the bootstrap values based on Neighbor joining/Maximum parsimony methods, respectively. Only the values greater than 50% are shown. Sequences are identified by the subspecies name, origin and country name, followed by the GenBank accession number (NK, North Korea; SK, South Korea). The mitogenome generated in this study is marked in bold face. (DOCX) [file pone.0207662.s011.docx]

**
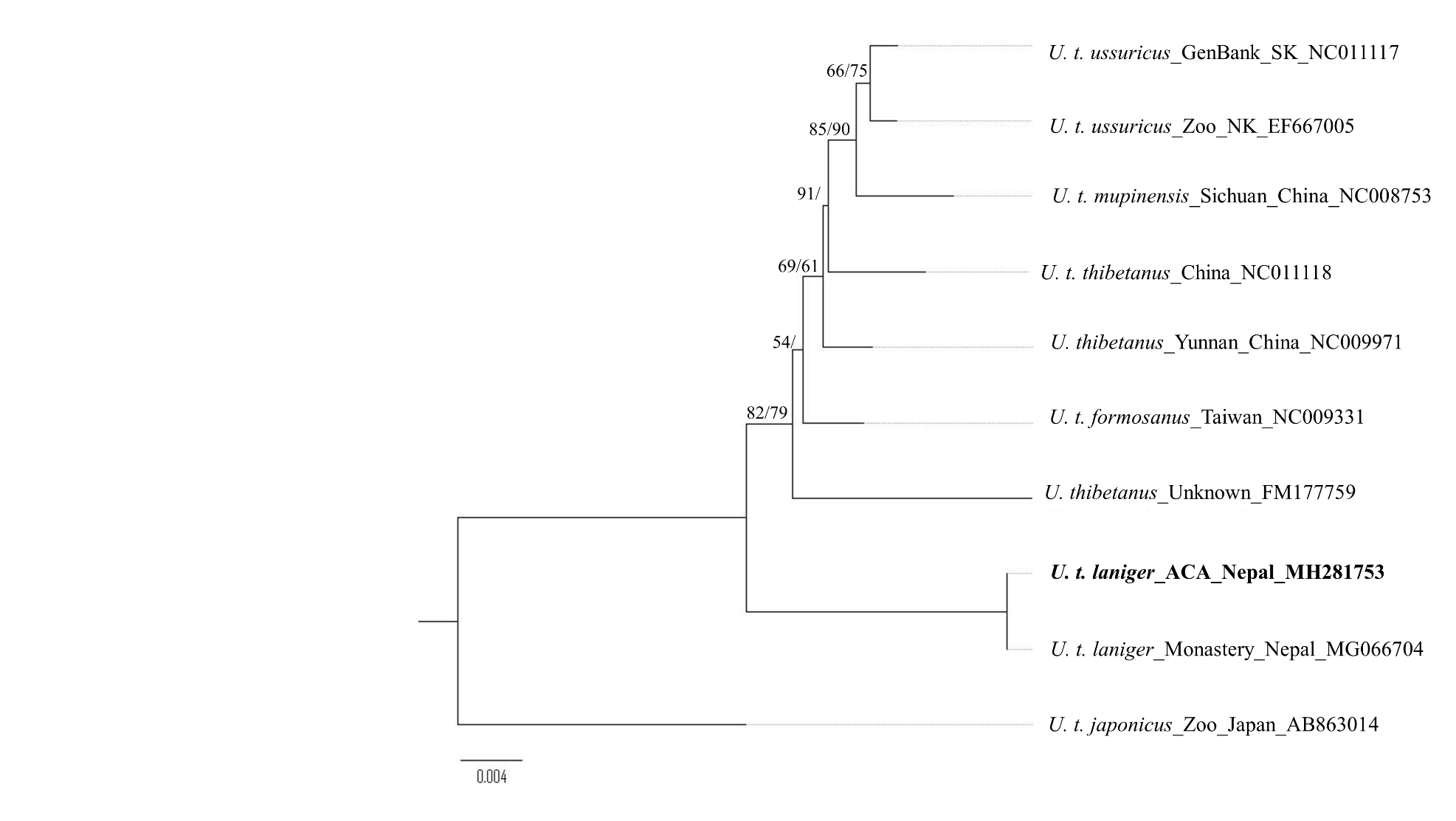
S3 Fig. Phylogenetic tree based on the complete cytochrome b gene (1,140 bp).** The numbers denoted at the node are the bootstrap values based on Neighbor joining/Maximum parsimony methods, respectively. Only the values greater than 50% are shown. Sequences are identified by the subspecies name, origin and country name, followed by the GenBank accession number (NK, North Korea; SK, South Korea). The mitogenome generated in this study is marked in bold face.
